# Should We Genetically Select for the Beauty Norm of Fair Skin?

**DOI:** 10.1007/s10728-017-0341-y

**Published:** 2017-03-21

**Authors:** Herjeet Marway

**Affiliations:** Department of Philosophy, University of Birmingham, Edgbaston, Birmingham, B15 2TT UK

**Keywords:** Beauty, Fair skin, Race, Colour, Discrimination, Genetic selection, Procreative beneficence, Procreative autonomy

## Abstract

Fair skin is often regarded as a beauty ideal in many parts of the world. Genetic selection for non-disease traits may allow reproducers to select fair skin for the purposes of beauty, and may be justified under various procreative principles. In this paper I assess the ethics of genetic selection for fair skin as a beauty feature. In particular, I explore the discriminatory aspects and demands of such selection. Using race and colour hierarchies that many would find objectionable, I argue that selection for beauty that is underpinned by such hierarchies is not a trivial selection. Given this, I claim that we should not make such selections.

There is a vast literature on the beauty norm for fair skin (e.g. [23, 30–33]) and some, though surprisingly less, literature on race and reproductive technologies (e.g. [4, 8, 22, 50–52, 61]). Much of the former has concentrated on sociological aspects of the debate and much of the latter on choosing gamete donors by racio-ethnic group. In this paper I take a different direction and develop ethical analysis on genetic selection for fair skin as a beauty feature.^1^ I draw upon issues at the intersection of race, colour, reproduction and justice in my ethical analysis. Because beauty is often trivialised and race rarely is, I seek to uncover ethical concerns of race and colour (that many will recognise as significant) through the vehicle of beauty (that many will regard as less so) in order to emphasise what is unjust about genetic selection for fair skin as a beauty norm. In particular, it is a non-trivial race and colour claim about discrimination and demand made through that of beauty. When race and colour underpin selection for beauty in these ways, such selection is anything but trivial.^2^

I proceed in four parts. First I provide some context and demonstrate that fair skin is a beauty norm for many, though of course not all, African-American and Indian women. I outline the race and colour hierarchies involved and the arbitrary distributive inequalities that may motivate conformity to the beauty norm. Second I indicate what demands the norm makes on this subset of women now through skin-lightening and what it might entail in the future via selecting lighter-skinned embryos. I trace the procreative principles that some might appeal to justify such selection too. In the last two parts, I centralise issues of justice that may counter these justifications. In part three I examine discrimination and ask what is wrong with fair skin selection *for* itself and then *for* beauty. I argue that the former naturalises social hierarchies of race and colour and the latter, once seen through this lens, may be unjust in intent and outcomes. In part four I discuss additional demands on reproducers and women in general. I submit that these technologies may result in greater pressure on the women to select embryos for fair skin, or a greater normalisation of and pressure to observe fair skin as a beauty norm. My claim overall is that we should not select embryos for the beauty norm for fair skin because to do so is not simply an insignificant choice but one that is discriminatory and demanding along racial and/or colour lines.

## Fair Skin as a Racialised or Colourised Beauty Norm

There are multiple beauty norms (norms relating to physical attractiveness) affecting various people. I consider just one: the norm for fair skin and its relation to women of colour in particular. I will focus on the examples of African-American (while there is huge variation in skin tone and it is somewhat imprecise, I use ‘black’ interchangeably in parts of the paper) women in the US and (with the same caveat, I use ‘brown’) women in India before I go on to discuss what problems of justice they might raise. It is important to note that the norm does not affect only women,^3^ only women of colour,^4^ or only these women of colour,^5^ but given space constraints these are the women I will consider. This is in part because much relevant research has been carried out on these women and in part because they are from relatively developed and developing to middle-income countries so provide an interesting comparison.

In both the Indian [2] and the American [7] contexts, there is a strong association between light skin and beauty and conversely dark skin and unattractiveness. In India, for instance, many women and men across religion, caste, age and occupation regard fair skin as a common standard of beauty and aspire to it [38]. In one study [42] involving 100 diverse students with a mean age of 22, some 78% of males and 63% of females included the words ‘light’ or ‘fair’ when describing prettiness (742-3), though several expressed concerns about this ideal. In the US, too, fair skin is connected to beauty among African American women [23]. In a study [10] involving 66 female black students with a mean age of 21.7 at a southeastern university, 70% thought that lighter skin was perceived as more attractive, and lightness was idealised by the women, except for those with the darkest skin tone. Though the briefest of snapshots, this is indicative of fair skin being a beauty feature in these contexts.

This is not to say all women in these contexts are subject to the norm or have a desire for fair skin, nor that women of colour are non-agential. For instance, black women (and judges) in beauty contests who aligned themselves to the Black Power Movement in the 1960 s and the politics of Black Is Beautiful would not subscribe to the norm [14]. Likewise, Indian women who are part of the current Dark is Beautiful campaign by Women of Worth which launched in 2009 do not [42, 66]. Further women may adopt the norm but appropriate it as entrepreneurs (e.g. Annie Turnbo Malone, who developed fair skin products for other African-American women without utilising bleach—[23]) or work against it by becoming vocal role models (e.g. Nandita Das, a Bollywood actress and ambassador for the UNFair campaign—[33]). This demonstrates a great deal of agency. Thus, women are not passive recipients of the beauty norm; some reject, some embrace with varying degrees of reservation and acceptance, and some complicate it. My claim is merely that fair skin is a norm for many women in these contexts, however they negotiate it.

There are discriminatory racial and colour hierarchies that underpin this connection between fairness and beauty and darkness and unattractiveness—and also between a ream of other positive (cleanliness, chastity, virtue and purity) and negative (dirtiness, promiscuity, viciousness, sexuality) traits respectively [2, 7]. By racial hierarchies I mean hierarchies (of subordination and domination) along some dimension (social, political, economic, etc.) in particular contexts between racial groups, where such groups are demarcated physically by features like skin colour, hair type, nose shape etc. [28]. By colour hierarchies I mean a parallel notion: hierarchies along some dimension in particular contexts between lighter and darker skins (e.g. [31]). Though these hierarchies, and the racism and colourism (e.g. inferiorisation or antipathy because of one’s race—[9]—or colour) often associated with them, can occur independently, they are connected in important ways [31]. For instance, in the US slave owners believed darker-skinned Africans were physically stronger but less intelligent than their lighter-skinned counterparts, and that mixed-race individuals (including those who were the offspring of slaves raped by slave owners) were more intelligent still. Those with lighter-skins were sometimes handed different roles because of this assumption, and gained more valuable skills than those with darker-skins [5]. Not only this but there was elitism within the black community, including the infamous Paper Bag Test (where anyone darker than a brown paper bag was not permitted entry to certain clubs, bars and churches) [23] and blue-vein aristocracy (where marrying other light-skinned people, light enough so their veins were visible, was encouraged) [10]. In India, there are various explanations, both pre and post colonisation, put forward for why fair skin is esteemed, including race, class, caste and geographical origin [33], but importantly race is not absent. For instance, light skin has been associated with Aryans who migrated into India from the north as well as with the British during colonial times [23]. Both fairer skinned groups had more power and privilege over the darker skinned Dravidian (e.g. the Aryans acquired or came to own agricultural land which became a source of power) or Indian group (e.g. the British ruled over and instigated divisive policies including favouring lighter skinned Indians for jobs) [42]. This both reinforced existing preferences for fair skin and exacerbated them. Thus in both the Indian and US context, race (and racism) and colour (and colourism) are connected, and often in complicated ways.

Moreover, there are strong reasons for having fair skin given the sorts of advantages that might accrue to fair skinned women in these contexts today. Though space constraints limit the discussion of all of these, two sorts of advantages serve as an illustration: (1) the sort of educational and career opportunities one has and (2) the sort of partner one might attract (e.g. [30, 31]). Both of these can significantly affect women’s lives depending on their skin colour and perceived beauty.

The importance of women having access to good jobs and education to better their lives and become financially independent is clear. In the US, Verna Keith and Cedric Herring [35] have shown that skin tone directly affects the level of educational attainment and occupational status of women: although darker skinned women in their study were employed, for instance, they were in less prestige jobs than fairer skinned black women.^6^ Margaret Hunter [30, 31] similarly argues that lighter skinned black women have higher educational attainment and are better rewarded for work. Keeping similar background characteristics constant, for every grade increase in lightness from 1 (*darkest*) to 5 (*lightest*), educational attainment increases by 4 months (a total increase of 1 year between women at either extreme of the scale), and income increases by $673 annually (a total difference of $2600 between the extremes) [30, 182–183].^7^

In India a recent phenomenological study by Sims and Hirudayaraj [59] explored the effect of skin tone on Indian women’s career aspirations and opportunities. It involved six women aged between 30 and 53 born in India, three of whom continue to live in India and three of whom live in the US.^8^ Darker skin was reported to affect choice of profession: “there is self-censorship with some girls. Girls do not apply to be in plays because they expect that they will not be chosen in lead roles” [59, 47] because of their darker skin. It also affects the jobs and opportunities that are available: “we don’t see any dark females as air hostesses…I don’t think people prefer dark-skinned people as front desk people” [59, 48].^9^ The beauty norm for fair skin does appear to limit career goals and opportunities for both Indian and African American women.

The second illustrative example is attracting mates, which is important in patriarchal contexts, such as where women cannot be propertied without being married [34] or where marrying well is a way to overcome poverty [32] or reach a higher status [30]. In general, fairness commands a premium and leads to greater marriage prospects in India, though it is not the only factor (e.g., a higher caste can have a bearing on marriageability, making skin tone one significant element against others—[42]). Analysis of matrimonial adverts on the *Hindustan Times* and *Times of India* websites, for instance, reveals that fairness for women (whether they are seeking a partner or are being sought) was a constant theme, no matter the caste or religion of the families and was as important as education [42]. Darker-skinned Indian women tend to be far less successful on contemporary Indian online dating sites too—in fact their successes compared to lighter-skinned Indian women in one study are described as “non-existent” [34, 65].^10^

Darker-skinned black women in the US are just as likely as lighter-skinned black women to be married, but they are less likely to have access to higher-status (understood as well-educated) partners than their counterparts [30, 31]. According to Hunter, even when they are otherwise comparable (in educational attainment, income, and parent’s education) a black woman’s spouse completes a further 0.28 years of schooling with every grade increase in skin lightness (a total of 1 year difference between the very fairest and darkest on the scale) [30, 186]. However, other factors, like a woman having a higher class or education level despite her darker skin tone, could mitigate the perceived negativity of darker skin, thereby complicating the picture [30, 190]. Monk [44] corroborates the general finding about fairer skinned women having access to higher status partners, though for him the difference is slightly less.^11^ In both the US and Indian context, skin tone matters for the sort of partner a woman might attract.

It is not merely having fair skin that generates advantages. Having straighter hair or lighter eyes *and* lighter skin in some contexts would bring more benefits than lighter skin alone, and other factors like education or caste in others can mitigate having darker skin. Yet light skin does have its benefits, such as getting a better job or a better partner. This affects one’s social position and prospects and could be an issue of socioeconomic fairness. Underwriting all this is a beauty norm for fair skin that exists in particular places and which arbitrarily values particular races or colours because of a history of racism and colourism—ultimately this is prejudicial.

## Attaining Lighter skin: Current Cosmetic and Future Genetic Techniques

Given the advantages of fair skin, it is no surprise that women are encouraged, and want, to lighten their skin to attain some of it. The point, again, is not that all women in these contexts aim to do this or are unhappy with darker skin, but that more are likely to want to lighten than darken.^12^ In what ways might skin lightening be encouraged and attained? There are answers both now and in the future.

### Now: Skin Lightening

Messages about lightening the skin are pervasive for Indian and African-American women. Female family members often stress the importance of fair skin: “parents and grandparents often comment on dark skin as a misfortune, disadvantage, and disability” [33, 67] especially with regards to marriage in India. Such relatives may encourage girls to use saffron, turmeric and creams to lighten the skin, warn them about dark skin preventing them securing a good husband, and show favouritism of lighter over darker skinned daughters [42]. The skin lightening market, including creams, soaps and ointments, is vast (61% of the Indian dermatological market comprise such products [66] including Indian brands like Fair and Lovely and increasingly offerings from Western brands such as L’Oreal) and it is lucrative (worth around $432 m) [3].^13^

While in the US there can be strong black pride sentiments in African-American families, there is often a similar narrative about fairness that comes from mothers, grandmothers and aunts. This includes, for instance, messages about not playing in the sun, encouragement to marry light or suggestions to bleach the skin (where mothers, grandmothers and aunts are lighter-skinned), or having lower expectations of darker skinned siblings (where those other family members are darker skinned) [64]. There are also several products marketed at African-American women, including Ambi Fade Cream, Black and White Cream, and Nadolina [23, 40].^14^ The messages one hears growing up and the products available can perpetuate the ideal of fairness equating to beauty.

Skin lightening products may, however, include harmful chemicals, and so are not without health risks. Those products with mercury, for instance, have serious physical risks including neurological damage, kidney disease, and a reduction in skin’s resistance to bacterial and fungal infections; psychological risks including anxiety and depression; and environmental risks including mercury eventually being discharged into wastewater and entering the food chain through fish, which can affect pregnant women especially [66]. Those products without mercury can have different harmful effects, but harmful effects nonetheless. Those with hydroquinone can lead to damaged skin and ochronosis, and those with corticosteroids bring risks such as eczema, infection, Cushing’s Syndrome, and skin atrophy [23], and it is often harder for women to stop using non-mercury products as this can lead to withdrawal symptoms (e.g. instant flare-ups of rashes) [17].^15^

### Future: Selecting for Fair Skin Through Genetic Reproduction

For reproducers undergoing IVF or artificial insemination, it is possible to select gametes of a particular race or ethnicity. Indeed it is not just possible; it is the primary criterion for selection of gametes in the US [22] and globally [4]. Within these racial or ethnic groupings, there are also classifications of gamete donors by skin tone. Though there is variation across banks, in one egg donation clinic in the US, these include fair (Caucasian), medium (mixed, Latino, some Caucasians), olive (Mediterranean, Southeast Asian, Latin American) and black (African) [61]. This offers reproducers the chance—though perhaps only a tenuous one [61], as I will discuss—to have a light skinned child.^16^ With pre-implantation genetic diagnosis (PGD) where embryos can be, or it is assumed (for the purposes of philosophical debate) will eventually be able to be, tested for disease (like Down’s Syndrome or mitochondria) *and* non-disease (like sex, intelligence or height) traits, it may be possible to extend this to more precise colour tests.^17^ A final PGD test on the embryo before implantation could assess the extent of fairness. So gamete selection may offer *some* (albeit sketchy) indication but we might think embryo selection adds a little more predictability.^18^ In other words, the test could enable reproducers to select an embryo amongst several embryos with light*er* or light*est* skin for the purposes of attaining standards of beauty for their children.

At this point, it is worth acknowledging that there may be scepticism about the availability, cost and desire for reproductive technology by women of colour in general and, by extension, for fair skin selection in particular.^19^ However, given that there are assumptions that fair skin is more beautiful and there are socioeconomic benefits to having fair skin, I suggest that selection may be plausible for some women. In particular, rather than simply lighten skin after birth (an ongoing and, given the chemicals involved, risky process), perhaps reproducers may be motivated to select a child for fair skin (a one-off process, though of course maintenance, like avoiding too much sun, is still required).

Selection for fair skin as a beauty feature may have two procreative justifications. One is procreative autonomy or liberty to decide not only whether to have children and when to have them [20], but—given this strong presumptive right—to decide on the sorts of children reproducers want [53]. This includes children with traits that they value, which are picked out in selection—such as, for present purposes, a beautiful child with fair skin. John Robertson, for instance, argues first that any genetic trait can be determinative of a reproducer’s decision to procreate at all. The likely import of such traits to one’s decision may vary: some traits (severe, untreatable, genetic disability) may be more central than others (eye or hair colour) to a parent (because they can not look after the child, it does not fit with their ideal family, or they want what is socially desirable). However, given that any trait can be determinative, genetic selection as a whole should be protected under procreative liberty rights [53, 431–432]. Second he argues that the harm associated with selection is not so great as to warrant interference or prohibition of the strong presumptive right. There is no direct harm to the child (since it is better to exist than not) and indirect harms (such as to classes of persons) is not sufficiently compelling, though the state may offer counselling or withhold subsidies to limit it [53, 428].

The second justification for selecting embryos based on colour is procreative beneficence [54] and the desire to do the best for one’s child. This includes having the child with the most well being, such as—in our case—the child who is likely to get the most benefits because they are fair skinned and regarded as beautiful. For instance, Julian Savulescu argues that, if parents undergo IVF and PGD, “couples (or single reproducers) should select the child, of the possible children they could have, who is expected to have the best life, or at least as good a life as the others, based on the relevant, available information” [54, 415]. This principle gives reproducers a moral obligation in his earlier work [54] or significant moral reason in his later work [55] to select the embryo that is likely to have the most wellbeing. This obligation can be overridden, such as if the parents hold “competing normative reasons” [55, 278], like a commitment to fighting prejudice, harm to others, or the parent’s welfare. Where it applies, though, the principle includes selection for non-disease traits, like our example of skin colour for the purposes of beauty.

There are three points to note about these principles. First, beauty is trivial but may motivate selection. Robertson, for instance, suggests that selection for eye or hair colour—and, we can infer, skin colour—are not very important but may play a role in procreative decisions. Second, race is more significant than something like beauty and *perhaps* should not motivate selection. Though neither philosopher is insistent on this. Robertson advocates that states could disincentivise, though not ban, selection that harms classes (such as races). Savulescu [55, 290, footnote 60] highlights that fighting (skin colour) prejudice could, but need not, qualify as an individual reproducer’s competing normative reason to not select.^20^ Third, these views suppose that beauty and race are discrete. Yet we have seen (Sect. 1) that there are hierarchies and socioeconomic advantages relating to race and colour in the beauty norm for fair skin. In sum, on these principles, selection for beauty is likely to be frivolous when compared to race, though race is not especially weighty to forestall selection at all, and beauty and race are distinct.

In opposition to this, I argue in the rest of the paper that beauty selection is not as trivial as might be thought, especially where we see this through the lens of race or colour, and that ultimately this should limit such selection. I do this by paying attention to the discrimination and demands of embryonic selection for the beauty norm for fair skin. This analysis will provide grounds to not make such selection, even if one wants this for a child or if it would create more wellbeing for it. I will principally discuss women and social classes rather than the embryo or future child as I assume for the paper, for reasons well traversed, that there is no harm that comes to the embryo (it is too underdeveloped) or child (it is better to exist than not).^21^

## Discrimination

I start by considering what, if anything, is discriminatory about genetic selection for fair skin as a beauty feature. I mean to ask whether there is something discriminatory first with fair skin selection (as opposed to non-disease selection in general) and second with fair skin selection for beauty specifically (as opposed to for other reasons). My argument is that there are inherent wrongs with selection for race or colour and much of this underpins, even if indirectly, selection for fair skin for beauty. Beauty selection for race or colour is therefore discriminatory and non-trivial.

### Fair Skin Selection

Multiple reasons may be given for why selection of non-disease traits, like height, intelligence or sex, is discriminatory, including because they contribute to greater inequalities, the creation of genetic underclasses, poor resource distribution, prejudices to particular classes etc. Though I do not have space to outline all of these, I do consider why selecting for skin colour in particular at the genetic stage would be unjust. I argue this is because it makes race (and colour) more of a natural a category than it is, which is to give credence to the false biological explanation for race. Let us take race and skin colour in turn.

On race, Dorothy Roberts has argued that the preoccupation with reproductive technology in the US has principally come from whites as a privileged racial group and is not shared by blacks as a non-privileged group. She points out that “sharing genetic traits seems less critical to Black identity than to white identity” [50, 263].^22^ The fixation with having genetic children, she argues, is connected to ideologies of racial purity (that races are biologically real and can be delineated and so preserved) and genetic inheritability (that dispositions or behaviours tenuously associated with races, like intelligence or industriousness, pass through genes) [50, 51].^23^ These ideologies are incorrect. Race is a social category and one that was created using false claims about biological difference between groups partly to justify narratives of superiority and racist policies, like slavery and colonialism. Given this, placing value on race, by preferring one race to another, in genetic selection of (e.g.) gametes is racist *if* the expectation is that stereotypical racial dispositions or behaviours can be conferred to one’s child [8, 33]. (I explore other reasons for selecting in the next part). Suggesting that race can be genetically selected, then, is giving false credence to the racialised claim about these biological differences and inheritability, and is discriminatory in contexts like the US with its history of racism.

Skin colour, on the other hand, is a physical feature of the body with some genetic predispositions to how fair to dark one might be, though as a phenotype skin colour is not static or immutable.^24^ Does that mean that selection for skin tone (a partly biological feature) is not as problematic as for race (a social category)? That the former can be selected for purely physical reasons absent of racialised baggage? I believe not. However, before I get to this, it is worth pointing out two caveats about relationship between race and skin colour, and the biological and social. The first is that skin colour, especially in heterogeneous populations like the US, is a prominent feature (though by no means the only one, since other features like hair type and eye shape are indicators too) of the social grouping of race. So phenotypic colour and race are not entirely unlinked. In the genetic case, as Thompson [61, 131] notes about egg donation, many might believe that the skin tone of the donor might transfer to the child in a way that correlates to social categories of race. Even if one thinks skin colour as a physical feature is conceptually distinct from race as a social category, they are, as I have suggested, importantly intertwined in some reproducer’s decisions in the real world. The second is, as I have shown, skin colour hierarchies exist in the US and India and these too are a social construct based on a physical marker of the body. Markets in eggs, for instance, reveal that some donors (predominately white or light-skinned) are paid a premium over others because they, in part, have the sorts of skin colours that are socially desirable [4, 22, 61].^25^ Thus there are social hierarchies relating to physical skin colour. If one adds to this that fair skin is superior and that associated dispositions and behaviours, like cleanliness or virtuousness, can be bequeathed to the genetic child, this sounds similar to what is problematic about selection for race. These two preliminaries are intended to highlight the complicated relationship alluded to earlier about race and colour and that there are social hierarchies attached to the physical marker of colour, either in itself and/or through the close connection to race.

Getting back to the original point: the suggestion that selecting for skin colour is (by and large) not discriminatory in itself if it is done for purely physical reasons, though selecting for race (by and large) is because it is social and assuming it does not have purely physical reasons.^26^ In addressing this I consider the main justification of skin colour selection: donor and intended parent phenotype matching so that it appears that the resulting child is naturally conceived.^27^ Berkowitz and Snyder [8] defend what they call Reasonable Phenotypic Approximation (RPA), but not race selection, arguing that it is not racist. Rather it “provides the infertile couple (e.g. a Caucasian male and Hispanic female) a child whose skin colour best resembles their own…what nature would have provided had the couple not been infertile” [8, 37]. Selection of gametes for skin colour (where Berkowitz and Snyder equate skin colour to race in this part of their discussion) for physical appearance, rather than the superiority or inheritability of dispositions or behaviours related to skin colours (that is races), should occur and would, in fact, prevent racism in reproduction in their view [8, 35].

Though Berkowitz and Snyder recognise that placing value on skin colour is integral to racism, their claim about naturalness, and biology that is implicit in the language of naturalness, speaks directly against Roberts’ concerns about racial purity in particular (and so seems to not limit racism in reproduction). Phenotypes may differ greatly *within* families of the same parentage, as Roberts [50] has argued about African-Americans and Mishra [42] has noted about Indians, and so the focus on preserving the ‘natural’ skin colour of a child, as though there is one, is suspect. Given this, the idea of matching donors for race, and I argue even skin colour, falsely reiterates the naturalness of each, and that there are the right kinds of gametes or embryos for parents to select. For instance, in terms of race, as Amrita Banerjee argues, whether by reproducers or physicians, “Racial matching via the ‘proper’ kind of eggs…implicitly pushes forward the troubling belief in the biological origin of race” [4, 123]. That there is the right kinds of egg for this race that differs from that race. Likewise, in terms of skin colour, reproducers may recognise the fluidity of racial categories but sometimes insist on more rigid colour categories in sperm selection, where it is to look like them [49].^28^ Yet skin colour matching implies both ‘proper’ kinds of gametes, when there are high degrees of variation, and that there are biological origins of skin colour that ultimately connect to race. The aim of RPA is to stop any value being attached to race by only permitting approximate natural skin colour selection. However it also assumes there is a right sort of donor to have and that, in effect, white or light-skinned donors should be used for white or light-skinned parents (as is the trend). Colour purity parallels racial purity, and (*if* there are also expectations about dispositions or behaviours passing to children) genetic inheritability.^29^

Berkowitz and Snyder conflate skin colour and race but, in so doing, this also demonstrates the similarities between them. Selecting for skin colour solely as a physical feature still has resemblances to selecting for race as a natural property and ideas of racial purity, which are false. There is an underlying assumption about the naturalness of each and that these can be passed to one’s offspring.^30^ This is especially troubling given the history of racism that uses a biological explanation to justify difference, and when colourism in its contemporary form is closely related to racism or (if one wants to keep race and colour distinct) has independent social hierarchies. It is geneticising and valuing skin colour and race, both of which are endemic in the history of racism, that make fair skin selection discriminatory. If this analysis is correct, I take it that race or colour selection for itself is inherent discriminatory; a wrong that many people would accept as such.

### Fair Skin Selection for Beauty

What about selection for fair skin for the purposes of beauty? To examine this, I outline first the significance of intent and unjust contexts before second considering outcomes. My claim is that once we uncover the race and colour dimensions of the beauty norm for fair skin, it is difficult to deny discrimination in skin colour selection for beauty. The selection is not inconsequential.

#### Intentions

Selection for fair skin for beauty could differ from selection for race or colour per se because of intent or lack thereof. Selecting for beauty would be wrong if the intent was racist or ‘colourist’—if selection was because of superiority of one race or colour or related assumptions about dispositions or behaviours passing to children. If it happens that it is not—e.g. if one thinks fair skin is beautiful, absent of superiority or expectations about dispositions or behaviours, simply because it is so—then this would not have the requisite intent. This is similar to non-beauty arguments about intentions and expectations when selecting for colour or race and is part of the justification for RPA [8, 22].^31^

Contrary to this, I argue first that the absolute lack of intent may be quite unlikely but second, even if it happens, that we should be cautious about such decisions as they can underwrite discriminatory patterns. First, I suggest that selection for fair skin in the pursuit of beauty does include *some* ideas about what is better and what benefits might accrue to one’s child that are rooted in some of the problematic ways discussed. In the Indian and African-American contexts, for instance, where there are social norms about fair skin and beauty, hierarchies about what is better or worse and associated (dis)advantages (i.e. colourism) do exist, and a propensity to lighten rather than darken skin is more likely at least in part because of this, as noted in Sect. 1. Now it is not necessarily the case that individual reproducers will select to a significant degree because of these reasons. Physical skin tone could be picked with miniscule notions of hierarchies or stereotypes being associated. However, what is important is precisely that there are *some* aspects of this that are relevant. Reproducers could select for beauty without appealing to such hierarchies and stereotypes but I find this implausible in a highly raced or ‘colourised’ context, which is, in part, what makes such a choice meaningful.^32^ In this regard, (race or) colour hierarchies not having *a* role (the question of relevant degree is up for debate) in selection in a racist and ‘colourist’ world are unlikely (but certainly not impossible). Rather than assume the intent is absent, this view suggests it is an open possibility. If the beauty norm for fair skin is selected for, in part, because of the discriminatory hierarchies and stereotypes associated with race or colour then such selection is not insignificant.

Second, imagine, though, that the requisite intent *is* missing on the part of reproducers (they do not resort to superiority or stereotype thinking when selecting for fair skin for beauty). Is selection for beauty here discriminatory? Here I am concerned with seemingly trivial individual decisions, such as some might think about beauty and fair skin selection, and the cumulative patterns of these decisions in particular contexts. Physicians (often motivated by promoting patient autonomy or beneficence towards the patient) and reproducers (perhaps governed by procreative liberty or beneficence) use a technology like PGD on a case-by-case, individual, basis. Yet the worry is that the extent to which the sum of those individual decisions, including for fair skin selection for beauty, maps onto and contributes to broader patterns of social discrimination, such as on darker skinned persons, would rarely be considered.^33^ In sperm selection, for instance, Quiroga [49] has noted how practitioners aim to genuinely help their patients (and so the thought for us is that they are not necessarily prejudiced), but that, in their advice about which sperm to select, they use and continue to underwrite racial classifications and ideas about racial purity and genetic inheritability. In embryonic selection for beauty for fair skin, one might make the decision without any intention about races or colours being better, such that many would sideline this as an unimportant choice. However, it still has meaning in a context of broader discrimination against darker skinned persons. Perhaps such selection would ultimately not matter in a genuinely racist or colourist free world. Yet the US and India are not such societies and decisions occur within and against these backdrops. This includes selection for fair skin for beauty, which I argue reproduces longstanding patterns that are discriminatory to certain classes of people.

#### Outcomes

Beyond intentions, outcomes related to selecting fair skin for beauty may be discriminatory. Setting aside reservations about the takeup of the technology, one possible outcome is permanent and predictable change to skin colour individually and overall. While lightening the skin through creams, soaps or lotions can have powerful and dramatic effects and staying out the sun can help with maintenance, these practices are temporary and require continuous observance.^34^ With selection, however, there is greater permanency of fair skin for the individual who has been selected. In addition, given the assumed predictability of fair skin embryonic selection, we might see a broader outcome. Greater certainty of this technique could mean more people actually getting fairer skinned children, with the resultant harm of the loss of diversity of skin colours overall, than when using comparatively less certain partner or gamete selection.^35^

Those individual reproducers that value fairer skin for beauty and are committed to achieving it may think this permanence and predictability is a positive result: this achieves what they want once and for all and with greater accuracy than other means. However, again, contexts and meanings behind this permanent outcome are relevant. While it might not be that skin pigment is a good in itself worth preserving longer term for these individuals, the discriminatory (racialised or colourised) ethos behind lightening—that dark skin is unclean, ugly, or vicious—is a reason to challenge permanent skin colour change and selection for fair skin as a beauty feature more generally. While some may initially dismiss a loss of some skin colours for beauty as insignificant, it is perhaps more disconcerting if the connection to race or colour is drawn out. In particular, the implication is that certain races or colours would be eliminated.

If the effect we are more concerned with is the expressed message of disvalue to *existing* darker skinned persons, then there are possible ways to mitigate this. Selection for fair skin for beauty could arguably continue if more appropriate messages about those with darker skin were communicated (that they are not unclean, ugly, vicious etc.).^36^ Indeed, some might suggest that *any* existing person that has worse wellbeing (including lesser intelligence, athleticism, beauty or musicality) than another possible person should logically recognise that they should have been selected against [24, 65]. This would not disvalue them per se and would be true for any individual; this would be a non-discriminatory and consistent approach to selection. But what is important is not just what that message is (that any embryo with a potentially better life should have been chosen) but also how it is interpreted by those who are the target group of that selection (i.e. persons who are in a discriminated against class versus those who are not, or who have never have been, in such a class). Those with darker skinned bodies and who have already suffered social disadvantages may well be more sensitive to the sort of message that dark skin can be selected out permanently than suggesting the same for light skin. Messages can be interpreted and misinterpreted by the recipient, making this an unreliable barometer for gauging the effect of messages. However there are differences in the groups hearing it based on their experiences in particular contexts, such as darker skinned groups that are stigmatised in the US and India. Failing to recognise this fails to pay attention to the concrete, lived experiences of those groups. While one might discount fair skin selection for beauty as not being especially serious, when we consider possible outcomes that relate to race or colour, like permanent changes to colour or messages expressed to existing darker-skinned persons, the discriminatory concerns become more visible.

Selecting for race or colour per se is discriminatory since to do this emphasises the biological aspects of race, which is objectionable given the historical development of racial difference. Selecting fair skin for beauty may occur with or without intent about better races or colours, but even if it does not, selection occurs in racialised or colourised contexts, not neutral ones. In addition, there may be outcomes that are harmful, especially where it leads to permanency or greater probability of changes to an already stigmatised group. When viewed through the non-trivial lens of race and colour, selecting fair skin for beauty can be discriminatory—which gives us grounds to not select for it.

## Demands on Women

Since the theme of this special issue is about demands of beauty, I now turn to the demands of selecting fair skin for beauty. I explore first the demands on reproducers and second the demands on women who do not use the technology. I claim that these demands can be high and, given some of the concerns already outlined in Sect. 3, discriminatory.

First, with regards to demands on reproducers who make the decision to select, the issue of autonomy is relevant. I assume for the purposes of discussion that reproducers are competent, are not coerced or manipulated, and have not necessarily internalised oppressive norms.^37^ Reproducers (or indeed anyone) may still be subject to more subtle external pressures to conform from others when the technology is available, and it is this I focus on. This conforming may include selecting the fair skinned embryo in contexts where many prefer fairer skin as a beauty feature and where it has benefits. Since decision-making only occurs in contexts, not in a detached way that is immune to those contexts [39], contexts are an important aspect to consider since they may be more or less conducive to doing what reproducers want. Of course, not all of those contexts are problematic. I suggest it is only those that are unjust—to particular groups based on social discrimination—that may end up being particularly pressurising and so harmful for autonomy. Though beauty selection would often be regarded as unimportant and a free choice that reproducers can make for their children, recognising the gravity and non-triviality of the race and colour dimension complicates this.

Disability and gender theorists have discussed the implications that genetic technology can have for women’s choices in contexts where certain sorts of bodies are preferred over others. In standard prenatal tests, for instance, Shakespeare [56] has highlighted that, in the UK, the decision to abort Downs Syndrome foetuses (i.e. non severe disabilities) are not free from value-laden, anti-disability norms. A woman can be reluctant to choose to keep a foetus, not because she does not want it (e.g. if she feels she is unable to raise it), but because the information revealed by tests (that it has Downs Syndrome) brings with it pressure (e.g. condescending attitudes from loved ones, strangers or medical professionals) to abort.^38^ Preconception sex selection may have similar pressures, though at an earlier stage. Some women may recognise the gender injustices of preferring a boy child, especially in contexts like China or India, but they may nonetheless feel as though they must make such selections. Women who fail to provide a boy child may be seen as lesser in their communities and girl children may also regard themselves as disappointments [15].^39^ In either the disability or sex example, this pressure may be subtle or it may be obvious, but there is pressure—ultimately stemming from societal prejudice—on women to do ‘the right thing’ by selecting in a particular way.

Would pre-implantation selection for skin colour as a beauty norm follow suit? Assuming that such selection is likely to occur in prejudicial contexts and that fair skin is valued and beneficial as a beauty feature, as discussed earlier, it seems reasonable that some parents may pick and implant the lighter skinned embryo. While, in theory, this might be without any attention paid to social norms or rewards, given (now) mainstream views of persons as social and decision-making as contextually rooted [13, 45], this seems implausible. Rather, such selection, either on the grounds of parental choice or the future child’s wellbeing, is harder *not* to make when there are norms that reward lighter skin and where lighter skin is regarded as more beautiful in society. And it is harder *not* to do so when one is subtly, and sometimes overtly, encouraged by others, including family members, like aunts and grandmothers.

I certainly do not wish to argue that those who select for fair skin because of (non-coercive) pressure in prejudicial contexts are *non*-autonomous in their decisions, since all people have *some* pressures on them and many contexts are prejudicial for one reason or another. I do want to highlight, though, that such contexts can be harmful to greater autonomy realisation.^40^ Overall, acknowledging pressures on reproducers to select for the beauty feature of fair skin in race and colour discriminatory contexts and how this may be harmful to greater autonomy realisation reveals that such selection is not trivial.

Second, there may be demands on women who do not select for fair skin but live in a world where use of the technology for such selection (and so the attainment of fairer skin) becomes more normalised. Imagine that couples or individual reproducers do not select either because they cannot (e.g. because of financial limitations where such selection is privately funded, as is likely in the US and India) or will not (on principled religious or secular grounds) or need not (because they simply do not want children), for instance. Imagine too that colour selection is routine as a procedure despite their non-selection. Imagine finally that fair skin continues to be beneficial in terms of social advantages in the ways outlined in Sect. 1, so it is desirable by many (even if not all). In this scenario, the technological procedure enabling fair skin selection (along with the achievement of fair skin) is normalised and this makes demands even on women who do not select and on women in general. In particular, it is likely that ongoing processes of skin lightening, staying out of the sun, or marrying fairer partners still occurs—and may well be more intensified—for many of those not selecting and for many darker women in general.^41^ There may be demands on them to continue or to start lightening in other ways to avoid missing out on the socioeconomic benefits and opportunities associated with having fairer skin.

Of course, the scenario presented is speculative and we could imagine variations of it. We could suppose, though I think somewhat erroneously,^42^ for instance, that—given widespread use of the procedure—fairer skin becomes more common and less valuable and conversely darker skin more rare and more valuable. I return to this possibility shortly but—assuming for the moment that fairer skin stays the norm—not adhering to a particular beauty norm, *if* one sees its attainment as important, is an uncommon trend in beauty. Not conforming risks not being seen as ‘normal’ and can often lead to lower self-esteem if one subscribes to the norm. (Those that do not, or who are presented alternative visions of normal, are less susceptible to this. For instance, black women suffer less from low body self-esteem than white women in the US in part because they do not identify with the predominately thin white models and actresses in the mainstream media—[43]—though greater assimilation into mainstream culture may mean such ideals affect black women increasingly—[1]. Of course women do reject norms and adopt others all the time. However, moving away from what is normalised is hard to do, especially where rewards continue to be linked to that norm and especially where those making the changes have limited power in particular societies. This suggests that, where beauty is self-definitional, it can be highly demanding, and discriminatory contexts relating to race or colour show that this is especially problematic.

Returning to the variation above, despite my scepticism, what if we assume that the norm *does* change to darker skin? If fairer skin is less in demand, it is possible that over time those with fair skin would become fewer and also the more marginalised, less powerful, less desirable group. However, if this happens, it occurs in quite a different context and trajectory to that which we know: of racist and colourist societies in which dark skin is less desirable and less rewarded. It is not so easy as to say the dynamics would simply reverse since fairer skinned persons have not undergone the same history (e.g. of slavery or colonisation) as African-Americans and Indians, though it is not to say this group relationship could not alter for other reasons over a period of time. Assuming that fortunes may simply switch, however, is to obscure the race and colour contexts of our world.

Pressures on reproducers to select for the beauty feature for fair skin may be high and harmful for fuller autonomy realisation. Further, achieving what is normal (whatever that might be) is important for those who subscribe to beauty standards including for fair skin. This can be demanding in itself, even for those who do not select embryos for fair skin, and even if what is normal is not a static concept. Some may find such demands relating to beauty in general easy to dismiss, but the non-trivial discriminatory aspects of race and colour relating to the beauty norm for fair skin make this less easy to do.

## Conclusion

I have considered whether we should genetically select embryos for fair skin for the purposes of beauty. Issues relating to beauty are often regarded as unimportant but I have used the non-trivial injustices of race and colour that underpin this norm to reveal that such selection it is anything but. Selection for the beauty norm of fair skin is discriminatory and demanding. If we take these to be plausible, they provide reasons for suggesting that we should not make such selections in the pursuit of beauty.

## Footnotes

1 Thompson [61] is an exception in that she extends the discussion from race/ethnicity to skin colour specifically and mentions attractiveness as a motivation for egg donor choice (138). I want to expand this to a focus on beauty specifically and embryo selection.

2 In this regard, other racialised beauty norms (e.g. hair type or eye shape) may fit this broader argument though less racialised beauty norms (e.g. breast size or height) would require a different analysis. The latter may well still be problematic for other reasons (such as being sexist) though I do not explore that here (though selection for reasons of sex and disability in general—rather than beauty—are mentioned in part 4).

3 Beauty norms govern appearance for all human beings but my focus in this paper will be women. This is because beauty is often gendered—in a patriarchal society where women have historically been associated with the body rather than the mind, women often must do more to meet beauty norms, and are judged by others and themselves for their success or failure in meeting them [6]. Further, in a hetero-normative culture, being described as ‘beautiful’ or ‘pretty’ are feminine terms and it would be derogatory to describe a man in these ways. With regards to skin colour specifically, women who have lighter skin are judged more aesthetically beautiful (pure, innocent, delicate etc.), whereas men, who are expected to have darker skin (they should be rugged, tough, sexual etc.), are less so. That women must have fairer skin when compared to men applies across all racial groups, white and non-white alike [7]. Of course some men do lighten their skin too (and perhaps this is increasing) so it is not that the norm does not affect men at all. I want to suggest though that women are subjected to it more.

4 The norm for fair skin does not only affect women of colour. As Amina Mire points out, historically in the US, women classed as white from eastern and southern Europe but with slightly darker pigment would use skin lightening products to appear more like Anglo-Saxon women with their fairer pigment [41]. She further highlights that in contemporary South African, US and European contexts, skin lightening products have had two sets of users: people of colour to lighten their pigment and white women for anti-aging (removal of freckles and sun spots, and blemishes) [40, 41]. However Mire also notes the racialised language and ideologies in these products.

5 The norm extends to other places. This includes, but is by no means limited to, South Asian women in India [16, 38, 47], African-American women in the US [10, 11, 14, 30], black and brown women in Jamaica [12], African women in Nigeria [46], Tanzania [37] and Ghana [48], African and Indian women in South Africa [18], and Southeast Asian women in Japan [62], Korea [38] and Malaysia [26].

6 Note that contra this, Monk [44] argues that educational attainment and occupational status applies for darker skinned black men too, and that occupational status in particular does not apply as much to darker skinned black women.

7 Using quantitative data of almost 1310 African American (and 596 Mexican American) women [30] and later qualitative data from 26 open ended interviews with women from these groups at university [31], Hunter found statistically significant trends for black women along these measures. So significant is this difference in income based on skin tone that Hunter regards it as comparable to the differences seen in residence (urban residents earn $2500 more than suburban residents), age ($84 increase for each year of age) and education ($1183 increase for each year of education) in determining income.

8 The women have careers ranging from a Bollywood actress (Nandita Das), university programme directors, human resource consultant, child safeguarding director, and postgraduate student [59, 43–44].

9 It also affects career aspirations through poor self-esteem: “there are times when my level of confidence hasn’t been good…there were moments I have thought… I am not sure if I am the right kind of person to be there” (Manisha [59], 48).

10 Though online dating is a more contemporary practice, this trend tracks the higher status often afforded to fairer skinned girls and women in traditional arranged marriages and in the dowry system (where fewer gifts and money for the groom are needed to secure their marriages) [33].

11 Ellis found that partners had 0.19 years of additional schooling per fairness increment.

12 Not all women do want to alter their skin colour. For instance, 47% of women in Bond and Cash’s [10] study said they did not want to change their skin colour, a fact the researchers take some heart in. But it is still the case that 37% of the black women in the study wanted to make themselves fairer, while only 17% wanted to go darker. So, of those dissatisfied, more want to lighten than darken (883). Likewise, though Nandita Das’ campaign is admirable, it is about keeping what one has and certainly not about darkening skin.

13 So pervasive are skin lightening ingredients in creams that often women (and men) will use them without realising they are using a skin lightener [42]. The industry also attracts celebrity endorsements from actresses and beauty queens like Aishwarya Rai, who is often presented as the archetypal modern Indian woman [33]. This indicates a strong use of and demand for skin-lightening products in the Indian market.

14 There are also popular examples of women who have lightened their skin in striking ways (such as the rapper L’il Kim), and popular expressions on internet forums of wanting to look like fair skinned celebrities, such as Beyonce and Halle Berry [23].

15 While in many poorer parts of the world, stronger and harmful (but cheaper) chemicals (mercury, hydroquinone and corticosteroids) are used for black or South Asian women for skin-lightening, in more affluent parts of the world weaker or safer (and more expensive) versions are used for white women for anti-ageing (hydroquinone, alpha hydroxy acids and kojic acids). Mire argues, though, that the distinction in how these different sorts of women are regarded for using similar ingredients reflects racist ideologies: such ingredients in products marketed at white women and promoted as high technology and scientific are perceived as a rational choice, while these same ingredients in cheaper cosmetic creams marketed at women of colour are frowned upon as an irrational choice. Thus, even though white and non-white women may use products with different aims but similar ingredients, there is a distinction in the racial and colour meanings depending on which women using the products [40, 41].

16 While gametes might offer some indication of skin colour through the donor, for instance, this is much less than is usually thought. Thompson [61] highlights that the idea that the donor’s skin colour traits get passed onto the egg (more so than random egg selection or general partner selection), and so the child, is causally weak. Thompson highlights this is why clinics often warn buyers or recipients about “the indeterminacy of the relation between a donor’s qualities and the qualities of a future child to which the donor’s egg contributed” [61, 136], or why one African American donor posted pictures of her two children, one with fairer skin than the other, on the database because she felt these “more accurately suggested the idea of a range of possible skin colors that could be expected among offspring born from her eggs” [61, 141].

17 I recognise here that methods other than PGD, which is often related to disease, might be more suited to perform this non-disease, appearance-related testing, but I will use PGD for brevity.

18 I consider ethical issues with this notion of predictability later on. It is these latter considerations of embryonic selection that I will mostly consider in this paper, and in particular ethical aspects relating to such selection, since these have been considered less.

19 Roberts [50] has pointed out the mistrust of such technology given the history of enforced sterilisation or birth control of poor African American women and how black women were discouraged from using reproductive technologies despite their higher infertility rates [50, 51]. Though such technologies are more available to them now, Roberts notes that it remains a small rich elite that is likely to use them [52]. In India, infertility and not having a biological child is seen as a source of stigma such that reproductive technology is increasingly being used, though as a final option after less intrusive methods [63]. Similarly with the African-American example, though, it is more urban, middle to upper class, women who utilise it [63] because of the cost. I acknowledge that such technology is expensive and has had a chequered past.

20 While I agree with Savulescu’s thought that we should not be prejudiced, I have disagreements with his general view especially around consistency with non-racial prejudice and with the strength of the claim. I do not have space to outline this here but these are ideas I am exploring elsewhere.

21 Embryonic selection evades the possibly stronger ethical concerns some may have about harm to the child that arise either in prenatal testing on the more developed foetus (tests on embryos are carried out very early, such as when the embryo merely has 8 cells—[27]—or 100 cells—[19] or by taking additional cells from the embryo (cells are going to be taken during PGD anyway, so this test is on the same tissue) [54, 58, 65, 67]. While there may be ethical concerns about testing on a (post-conception) embryo rather than selecting a (pre-conception) gamete, I will assume there are no considerations of harm per se to the embryo: it being too underdeveloped to claim rights of personhood. Though this will be contentious for some, I will assume it is the case given a lack of consciousness. I am going to sidestep possible harms to the child.

22 Roberts has also argued that black women are less wedded to the idea of having children that are genetic copies of them, accepting instead that “there are often a melange of physical features—skin and eye colour, hair texture, sizes, and shapes—within a single family. We are used to ‘throwbacks’—a pale, blond child born into a dark-skinned family, who inherited stray genes from a distant white ancestor…We cannot expect our children to look just like us” [50, 263], and that this need for this genetic tie is a white racialised obsession.

23 On racial purity, note the one-drop rule in the US where any black ancestry at all classifies the person as black. On genetic inheritability, note the claims about (e.g.) intelligence being a white disposition.

24 The biology and passing on of skin colour is important here. As Thompson puts it about egg donation, “if skin tone is salient in choosing an egg donor, it reveals hope for or belief in some kind of biological preservation of skin tone” [61, 131].

25 Both Banerjee [4] and Fogg-Davis [22] highlight that these are predominately white or light-skinned donors, and Banerjee that this is despite the higher availability of darker-skinned eggs, though Thompson [61] believes in time, because of donor shortages, any donor of any skin tone will be used.

26 I say ‘by and large’ here because the reason might make a difference. For instance, if selecting skin colour is to increase diversity of phenotype in the family (which I discuss later) or selecting by race occurs in tragic cases of preventing racism (I do not have space to discuss this claim in this paper but am developing it elsewhere) it may well be permissible for Berkowitz and Snyder [8].

27 Intended parents may want a ‘plausible match’ either per se [61, 139] or to keep the use of reproductive technologies a secret from others (thanks to a reviewer who pointed this out), and having a child that most resembles them (including their skin colours) achieves these aims. I focus less on the secrecy aspects in this paper.

28 One can see this in a case study by Seline Szkupinski[49] of couples selecting sperm, and in particular Raeshell, who is (an unspecified skin toned) African-American, and her partner, Tom, who is light-skinned African-American. Though Raeshell recognises the fluidity of race and how it doesn’t matter what racial category the sperm comes from as the child will still be raised and classed as black (given e.g. one-drop rules from whites), she *does* want a lighter-skinned donor over a darker-skinned black donor so the child looks like her husband [49, 155–157]. This indicates that she sees skin colour as more fixed than she does race and so why matching is important to her. But skin colour hierarchies too are social constructs and about purity of the lighter-skinned persons within a particular racial group.

29 Matching in fact motivates the trend of typically having eggs from white or light-skinned donors for white or light-skinned parents—Banarjee notes: “despite the fact that eggs from women of colour are now available in increasing numbers within the international market, there is a distinct global preference for eggs from fair-skinned women” [4, 123]—while poorer non-white or darker-skinned women are typically used to gestate those eggs in surrogacy arrangements. Thompson, however, thinks this trend may change in time [61]. It also motivates the false assumptions that Roberts identifies about racial purity and genetic inheritability. With regards to embryo selection, if RPA was adopted for gametes, then parents could choose the fairer embryo from the ones that they have in front of them (as presumably the embryos would all approximate the reproducer’s phenotypes), believing it to be the sort of child they want or the one with the best life chances. This would be in line with RPA but it would still facilitate skin colour privilege (and arguably race privilege).

30 I discuss non-racist reasons in relation to beauty in the section below.

31 Possible non-prejudicial reasons here might be that one selects skin colour for purely health reasons in India (say in the health case where darker skin is better protected against melanoma—[55]—where this would be because of expectations of better health) or for diversity reasons in the US (say for phenotypic variance within a family—[8]—where the gains anticipated are only physical differences) such that there is no appeal to social hierarchies or stereotypes at all. Interesting, Fogg-Davis argues about partner selection and gamete selection: “Assisted reproduction, as the name suggests, brings reproductive decision making into public view. Racial choices made in this arena publicly reinforce and make explicit the routine use of racial discrimination in the choice of a partner for procreative sexual intercourse, It is not so much that the former is morally worse than the latter. Both operate on the level of racial stereotype, prejudging and weeding out certain individuals based at least partly on their ascribed race” [22, 14].

32 Stephen Wilkinson [65, 226] raises a similar point in relation to sex stereotyping about sex selection: though strange, one could select for biological features of a sex (ability for a boy to grow a beard, for instance) without socially stereotyping. However, if part of the reason for the selection is character traits that the particular sex might have (assuming that the boy will want to go fishing), then this is socially stereotyping.

33 This worry is similar to the well-stated concern about assumed post-racial or post-colour societies where it is believed that the discrimination problem is somehow solved with formal equality legislation. However, policies, institutions, attitudes, beliefs, associations etc., may continue to be prejudicial and there may be colour-blind discrimination if one assumes an equal playing field or is not vigilant to discrimination along race or colour lines. This may occur in our case of selection too, as I discuss in the main text.

34 More enduring changes are promised online by ‘skin lightening surgery’, but it is unclear whether this is surgery proper (in the way breast augmentation or eye shaping surgery is) or a combination of other ongoing treatments (such as laser surgery for removal of sun spots, creams and skin peels).

35 This point draws on arguments about certainty and sex selection through innocuous means outlined by Stephen Wilkinson [65]. Recall there is assumed certainty because though children may share the same parents, both African American and Indian families may have darker and fairer skinned children within them [33, 50], and while there is no guarantee of skin colour in gamete selection as is often declared by clinics [61], arguably embryo selection could enable more predictability, given it is post-conception and pre-implantation, than these other methods.

36 This follows similar concerns people might have about selecting against disability and the effect of this message on existing disabled persons. See (e.g.) [25, 57] for a disability equivalent claim about sending appropriate messages. Also the Fair is Beautiful campaign attempts to improve messages about darker skinned persons now, though in relation to ongoing use of temporary skin lightening products (and with varying success), and this might apply to permanent selection.

37 That is, they are competent and critically reflective (they can endorse desires), are not coerced or manipulated (this being obviously inimical to autonomy), and have not necessarily internalised norms (this bringing up a unique set of issues). Within the category of coercion, I might typically include a severe lack of financial means that pushes one in a particular direction, but I do not think this straightforwardly applies to the case of embryo selection given what I have said about relatively wealthier women using the technology. One might think it applies to a case of less affluent individuals using IVF and PGD as a one-off procedure given the cost.

38 For instance, a woman was criticised by her (supposedly nondirective) counsellor when she decided to keep her child: “What are you going to say to people when they ask you how you could bring a child like this into the world?” [29, 59]. In another example, a woman who decided to keep her baby after a prenatal diagnosis of spina bifida says: “every time there was a knock at the door or a letter we thought ‘here’s another lecture’. They kept saying things like it was wrong to keep the baby…it would be wicked…They meant well but…” [21, 118]. If the woman chooses not to have the tests, she may be judged as not only not doing the best for the child but that any subsequent difficulties she encounters raising the child are her responsibility. A woman who learns her new born daughter has Downs Syndrome says, “When I started to cry, I overheard him [the physician] say, ‘What did she expect? She refused prenatal testing” [60, 70].

39 Dai, a Chinese born academic in the US with an older sister, for instance, discusses how her parents having a boy child, instead of Dai, a girl child, as the second child would have fulfilled Dai’s mother’s role as a ‘good wife’, and the devastation and disappointment her mother and others felt at the ‘failure’ by delivering Dai. She relays her mothers’ and, despite her commitment to gender equality, Dai’s own sense of anticipation at the opportunities and possibilities that preconception sex selection as a technology will allow—to be the ‘good wife’ and avoid being the disappointment [15].

40 Feeling pressure can truncate a fuller realisation of what one wants (constrained choices, whether by time, money, inveiglement, etc., are one’s choices but we recognise them as constrained nonetheless). Feeling pressure in unjust contexts, whatever that injustice but where that certainly includes racialised and colourised injustices, is worse because it stems from a morally arbitrary/discriminatory feature to which one feels she must fold. Of course, many reproducers *will* resist and not select for fair skin, broader norms notwithstanding, and many will select for it when fully cognizant about broader norms, and be sure that they want do it nonetheless (plus many other possibilities besides). However this is not to say that there are no demands on them to do so where light skin is valued as beautiful and makes a life go better. The contexts we are discussing, at the very least, reward fair skin as a beauty feature—owing in part to prejudice—and, as noted earlier, the norm exists however one negotiates it. Pressure to choose, then, does not equate to non-autonomy, though it limits a fuller autonomy realisation. Yet we should acknowledge the societal prejudices towards those with darker skins that suggest they are less beautiful than those with fairer skins, and recognise that these are the contexts in which selection is made and in which pressure to select accordingly may occur.

41 Though—to be more nuanced—perhaps (in line with Bond and Cash’s [10] study) those with the darkest skin in the US and (in line with Mishra’s [42] study) those from higher castes in India will not feel this demand as much. Still, those that do feel it yet who do not select for fair skin may well decide they need to lighten their skin.

42 I think there are problems with this sort of scenario, however. One point (though it does not touch on demandingness per se) is that it does not seem as though dark skin in racist or colourist societies is valued in contexts where it is rarer. Aborigines or Native Americans are not more valued now that their numbers are fewer, for instance. (Thanks to Scott Wisor for this point). Given that there are no pressing reasons for the trend to go the other way—as some have suggested that disproportionate sex selection for boys would go given the eventual imbalance of girls to boys [54]—in racist or colourist societies, I am sceptical this would occur. Even if it did not correct itself, those with darker skin who are fewer in number might be more valuable but, in racist or colourist contexts (it is not clear these contexts would change), they might also be regarded as a commodity or as fetished, in the way darker skin has tended to be [7, 14]. This is similar to Neil Levy’s [36] claim that fewer women in sexist societies because of selection might be regarded as commodities rather than valued per se. Being commoditised or fetished in this way would be harmful as it fails to see the person in all their complexity and individuality. In support of the thesis of this paper, then, selecting fair skin for beauty is by no means trivial where commodification or fetishisation on account of race or colour occurs.
